# Phenotypic and genotypic antibiotic susceptibility profiles of Gram-negative bacteria isolated from bloodstream infections at a referral hospital, Lusaka, Zambia

**DOI:** 10.1371/journal.pgph.0001414

**Published:** 2023-01-31

**Authors:** Kaunda Yamba, Chileshe Lukwesa-Musyani, Mulemba Tillika Samutela, Christine Kapesa, Mudenda Bernard Hang’ombe, Evans Mpabalwani, Lottie Hachaambwa, Sombo Fwoloshi, Raphael Chanda, Mirfin Mpundu, Glory Kashweka, Ruth Nakazwe, Steward Mudenda, John Bwalya Muma

**Affiliations:** 1 Department of Pathology & Microbiology Laboratory, University Teaching Hospitals, Lusaka, Zambia; 2 Department of Disease Control, School of Veterinary Medicine, University of Zambia, Lusaka, Zambia; 3 Department of Biomedical Sciences, School of Health Sciences, University of Zambia Lusaka, Zambia; 4 Department of Paraclinical Studies, University of Zambia, School of Veterinary Medicine, Lusaka, Zambia; 5 Department of Paediatrics & Child Health, School of Medicine, University of Zambia, Lusaka, Zambia; 6 Department of Internal Medicine, Infectious Diseases Unit, University Teaching Hospital, Lusaka, Zambia; 7 ReAct Africa, Honnington Close, Greystone Park, Harare, Zimbabwe; 8 Department of Pharmacy, School of Health Sciences, University of Zambia, Lusaka, Zambia; Zagazig University, Faculty of Medicine, EGYPT

## Abstract

Bloodstream infections (BSI) caused by antimicrobial-resistant (AMR) Gram-negative bacteria (GNB) are a significant cause of morbidity and mortality. Third-generation cephalosporins (3GCs) have been used as empiric treatment for BSI and other invasive infections for years; however, their overuse could promote the emergence of extended-spectrum beta-lactamases (ESBLs). Thus, this study aimed to determine the epidemiological, clinical and microbiological features and the effects of antimicrobial resistance on the outcomes of BSIs at a referral hospital in Lusaka, Zambia. This was a six-month prospective facility-based study undertaken at a referral hospital in Lusaka, Zambia. As part of the routine diagnosis and patient care, blood samples for bacteriological culture were collected from patients presenting with fever and processed for pathogen identification and antimicrobial susceptibility testing using the VITEK 2 Compact instrument. ESBLs and plasmid-mediated quinolone resistance (PMQR) associated genes were determined using the polymerase chain reaction method. Patient information was collected using a structured data collection sheet and entered in CSpro 7.6. Data were analysed in WHOnet and STATA version 14. A total of 88 GNB were isolated, of which 76% were Enterobacterales, 14% *Acinetobacter baumannii* and 8% *Pseudomonas aeruginosa*. Resistance to third and fourth-generation cephalosporins was 75% and 32%, respectively. Noteworthy was the high prevalence (68%) of inappropriate empirical treatment, carbapenem resistance (7%), multi-drug resistance (83%) and ESBL-producers (76%). In comparison to *E*. *coli* as a causative agent of BSI, the odds of death were significantly higher among patients infected with *Acinetobacter baumannii* (OR = 3.8). The odds of death were also higher in patients that received 3GCs as empiric treatment than in those that received 4GCs or other (none cephalosporin) treatment options. Structured surveillance, yearly antibiogram updates, improved infection control and a well functional antimicrobial stewardship (AMS) program, are of utmost importance in improving appropriate antimicrobial treatment selection and favourable patient outcomes.

## 1. Introduction

Antimicrobial resistance (AMR) has emerged as one of the greatest public health threats of the 21^st^ century [1]. Globally, the economic impact of healthcare costs is predicted to increase in a range equivalent to US$300 billion—US$1 trillion each year by 2050, with a corresponding 10 million deaths annually by the same year [2]. In 2019, the Global Research on Antimicrobial Resistance (GRAM) study evaluated the global burden associated with drug-resistant infections and projected an estimated 4·95 million (95% UI 3·62–6·57) deaths, of which 1·27 million (0·911–1·71) were directly attributable to drug resistance [3]. Multi-drug resistance (MDR) is increasingly prevalent in clinically essential pathogens and threatens to hinder the treatment of diseases [4]. MDR has worsened the outcome of bloodstream infections (BSIs) or bacteraemias that are already a growing public health concern [5]. Enterobacterales such as *E*. *coli*, *Klebsiella pneumoniae*, *Enterobacter species* and other Gram-negative bacteria (GNB) such as *Pseudomonas aeruginosa* and *Acinetobacter* species have been implicated in hospital acquired infections (HAI)) and community-acquired infections (CAI) [6, 7].

Successful treatment of BSIs relies on prompt identification of the causative pathogen, antimicrobial susceptibility patterns and selecting appropriate treatment. The rise in resistance to beta-lactam antibiotics, driven by the production of beta-lactamases, is now a public health threat [7]. Most literature review studies done on the prevalence of Extended spectrum beta-lactamases (ESBLs) in Africa have found a high prevalence with varying incidence from country to country [8, 9]. ESBLs are plasmid-mediated enzymes resulting from point mutation of TEM or SHV β-lactamases that are widely distributed among the Enterobacterales. In recent years, several new ESBLs (CTX-M, PER, VEB, and the GES) lineages have emerged [10]. Although class C β-lactamases (*Amp*C) confer resistance to the same antibiotics as ESBL, *Amp*C activity additionally affects cephamycins and is not affected by ESBLs inhibitors [7]. Furthermore, *Amp*C-producing GNB act as a hidden reservoir for ESBLs; thus, the co-existence of these enzymes complicates the treatment of GNB as *Amp*C beta-lactams may mask the recognition of ESBLs [7].

Patients with infections caused by ESBL-producing bacteria have poorer clinical outcomes, increased hospital stay, higher hospital costs and can lead to sepsis. Sepsis is a significant driver of antibiotic use and is defined as a life-threatening organ dysfunction caused by a dysregulated host response to infection. According to surviving sepsis guidelines, it causes severe morbidities and mortality [11–13]. Poor clinical outcomes have been worsened by the co-existence of ESBLs and resistance to other classes of antibiotics such as aminoglycosides and fluoroquinolones. Carbapenems are the treatment of choice for serious infections caused by ESBL-producing organisms [14]. However, using carbapenems as first-line treatment would increase the hospital cost and facilitate the spread of carbapenem-resistant pathogens, thereby limiting treatment options for invasive infections and raising mortality [15]. Therefore, we hypothesized that third-generation cephalosporin-resistant GNB could contribute to BSI’s poor treatment outcomes at the University Hospital (UTH) in Lusaka, Zambia.

In low-or middle-income countries (LMICs), beta-lactams are widely prescribed as empiric treatment, with third-generation cephalosporins (3GCs) being the most commonly used antibiotics [16]. A 2016 study that looked at aetiology, antibiotic resistance and risk factors associated with neonatal sepsis at the University Teaching Hospital (UTH) in Lusaka, Zambia, found 3GC resistance to be above 95% [17]. However, a point prevalence survey conducted at the same hospital in 2018 still found 3GCs to be the most commonly prescribed antibiotics in hospitalized patients and prescription of 3GCs was at 57.9% [18]. Thus, this study aimed to determine the epidemiological, clinical and microbiological features and the effects of antimicrobial resistance on the outcomes of BSIs at a referral hospital in Lusaka, Zambia.

## 2. Materials and methods

### 2.1. Study site and design

A prospective study was conducted at the University Teaching Hospital (UTH) in Lusaka, Zambia from October 2021 to March 2022. UTH is a national tertiary referral hospital with a bed capacity of about 1,665, offering specialized care to referral patients. It is a highly specialized facility comprised of five hospitals, namely: The Adult Hospital, Children’s Hospital, Mother and Newborn Hospital, Eye Hospital and Cancer Disease Hospital (CDH).

BSI was defined by positive blood cultures in a patient with systemic signs of infection [19]. The patients whose blood was drawn for blood culture, gave consent and were willing to respond to the questionnaire were included in the study. Patients admitted to the CDH were excluded from the study as a result of restricted entry into the cancer wards that prevented access to the patients for consent and data collection. Patients from the Eye hospital were also excluded because patients were mainly attended to in the out-patient clinic. Two-hundred and six patients were enrolled from the included wards. Blood samples were collected from the patients for bacteriological culture as part of the routine diagnosis and patient care, and were processed for pathogen isolation, identification and antimicrobial susceptibility.

The data collection sheet was administered by trained clinicians, and data was entered in CSpro 7.6 and treated with confidentiality. The socio-demographic, clinical and empiric treatment data collected was age, sex, types of symptoms, on-set and duration of fever, history of past hospital admissions and antibiotics given as empiric treatment. In order to obtain data on patient outcome and duration of hospital stay, a second file review for all patients that had confirmed Gram-negative BSI was done 30 and/or 45 days, post-admission.

### 2.2. Sample size determination and sampling method

This was a facility based study based on routine analysis of samples submitted to the Microbiology Laboratory at UTH as part of patient management. Convenient sampling was therefore adopted with the aim of isolating at least 50 GNB causing BSI. This sample size was perceived sufficient enough to observe any AMR pattern and genetic diversity if present as described by Nagelkerke et al., 2015 [20]. Based on the 2017 to 2018 UTH Microbiology Laboratory reports on BSI, we expected to process an estimated 250 blood samples, to recover 50 GNB, assuming a conservative 20% recovery rate. All blood culture samples submitted to the Microbiology laboratory from October 2021 to March 2022 were analysed.

### 2.3. Specimen collection and processing

As part of case management at UTH, blood was drawn from patients presenting with fever or any other symptoms requiring a blood culture, this was done before antibiotic treatment was commenced [21]. Each culture bottle was inoculated with eight to ten mls of blood from adult patients, while the volume of blood drawn from paediatric patients was guided by body weight as described by Kellogg et al. [22]. The blood was inoculated in two (when available) or one automated aerobic blood culture bottle (BD), after which it was transported to the Microbiology Laboratory within the UTH. Collecting two or more blood culture samples from different sites helps to distinguish true bloodstream infection from contaminants. In resource-limited settings like Zambia, a positive result from only one blood culture is interpreted with the help of clinical presentation, such as fever and other signs of sepsis syndrome [23]. The blood culture bottles were incubated in the Bactec machine (BD Bactec FX, Wokingham Berkshire, United Kingdom) till they flagged positive or up to seven days for those that were negative. All blood culture samples that flagged positive had a Gram stain prepared and were sub-cultured on MacConkey, blood, and chocolate agar plates (Oxoid, Basingstoke, UK). Before sub-culturing, the blood culture bottle tops were disinfected with iodine to prevent the introduction of contaminants [24]. Iodine was the only available disinfectant at the time of the study and although the recommended ethyl alcohol is more superior to iodine, iodine has also been found to prevent contamination [24, 25]. MacConkey agar plates were incubated aerobically at 37°C for 24 hours, after which, the plates were examined for both lactose fermenters and non-lactose fermenters suggestive of GNB. Gram stain was performed to confirm the isolates were GNB [26].

### 2.4. Identification and Antimicrobial Susceptibility Testing (AST)

The presumptive Gram-negative isolates were then subjected to identification and antimicrobial susceptibility testing using the VITEK 2 Compact instrument (Biomerieux). This was achieved by using the VITEK GN cards for identification and VITEK AST-GN83 and GN86 cards for AST [26]. *Pseudomonas aeruginosa* is known to be intrinsically resistant to ceftriaxone and cefotaxime [27], hence the resistance to 3GC in *Pseudomonas aeruginosa* isolates was based on susceptibility to ceftazidime. Each batch of VITEK cards was quality controlled using *Escherichia coli* 25922 control strain. The isolates were then stored in glycerol at -80°C. AST data was entered into WHOnet 2020, then managed in Excel spreadsheets and exported to STATA 14 for analysis. The proportion of Resistance (R %), Intermediate (I %), Susceptible (S %), (MDR %), extensively drug-resistant (XDR %), pan drug-resistant (PDR %) were estimated using WHOnet analysis. According to the CLSI guidelines, the definitions of susceptible, intermediate and resistant were based on the CLSI interpretations [28]. MDR isolates were defined as resistance to at least one agent in three or more antibiotic classes, XDR as resistance to at least one agent in all but two or fewer antimicrobial categories (i.e. bacterial isolates remain susceptible to only one or two categories), and PDR was defined as resistance to all agents in all antimicrobial categories [29].

### 2.5. Molecular analysis

Deoxyribonucleic acid (DNA) was extracted using the Nuclisense easyMAG (Biomeriux). Fifty-five isolates resistant to both a 3GC and a fluoroquinolone antimicrobial were randomly selected and subjected to PCR using the Sigma-Aldrich primers (S1 Table). The following resistance genes determinants were screened; ESBL determinants (*bla*TEM, *bla*SHV, *bla*CTX-M), *Amp*C β-lactamase (*amp*C), fluoroquinolone resistance determinants [plasmid-mediated quinolone resistance (PMQR) genes *qnrA*, *qnrB* and *qnrS*) and carbapenem resistance determinants (*bla*OXA, *bla*NDM, *bla*VIM)]. The primer selection and PCR protocol used was based on Farkas et. al [30]. PCR reactions were carried out using the following protocol: 2 μL of extracted DNA was combined with 12.5 μL of 2X master mix, 1 μL forward and 1 μL reverse primer, and the mixture was made up to a 25 μL volume with nuclease-free water. The Veriti 96 Well Thermal Cycler-Applied (Biosystems, Pittsburg, PA, USA), was used for PCR amplification, initiated at 96°C for 1 min, followed by 35 cycles at 96°C for 1 min, annealing for 1 min, and lastly, extension at 72°C for 2 min. The final extension was at 72°C for 10 min. Positive controls (previously positive samples) and negative control (nuclease-free water) were included in each amplification reaction [30]. The PCR products (1/10 volume) were analysed by gel electrophoresis (Bio-Rad, Hercules, CA, USA) at 100 volts for 30 minutes using 1.5% agarose gels (BD Difco) in 1X TAE buffer (Tris-acetate EDTA). The gels were stained with ethidium bromide (Sigma, St. Louis, MO, USA), and the PCR products were visualized under ultraviolet light [31]. As shown in (S1 Table), a single band with amplicon sizes was observed.

### 2.6 Data analysis

Data were managed in Excel spreadsheets and analysed in STATA version 14. The variable age was grouped into three categories as follows: Neonates (≤ 28 days), Paediatrics (≥ 29 days to < 16 years), and Adults (≥16 years).

To facilitate the analysis, the continuous variable "Length of Hospital Stay (LOS)" was transformed into categorical variable with binary outcomes by grouping those who stayed less than 25 days (short stay) and those who stayed ≥ 25days (long stay). The potential associations between the hypothesised categorical explanatory variables and the dichotomous outcomes (Recovery or death) were assessed using Fisher’s exact test. Collinearity between explanatory variables was checked using Fisher’s exact test. Explanatory variables in a univariate analysis showing a p-value <0.20 from Fisher’s exact test were selected as candidate variables and taken into the multivariable logistic models. The multivariable model was built using a backward selection strategy, using a p-value of <0.05 of the likelihood ratio test as inclusion criteria. Model fit was assessed using the Hosmer Lemeshow test, *lfit*, *lroc* and *lsens* procedures in Stata for the logistic model.

### 2.7 Ethical clearance

The study was conducted according to the guidelines of the Declaration of Helsinki and approved by the Ethics Committee at Eres Converge institutional review board (Ref. No. 2019-Aug-017). The regulatory approval was obtained from the National Health Research Authority (NHRA) of Zambia. Informed consent was obtained from all participants involved in the study.

## 3. Results

**Fig 1:**
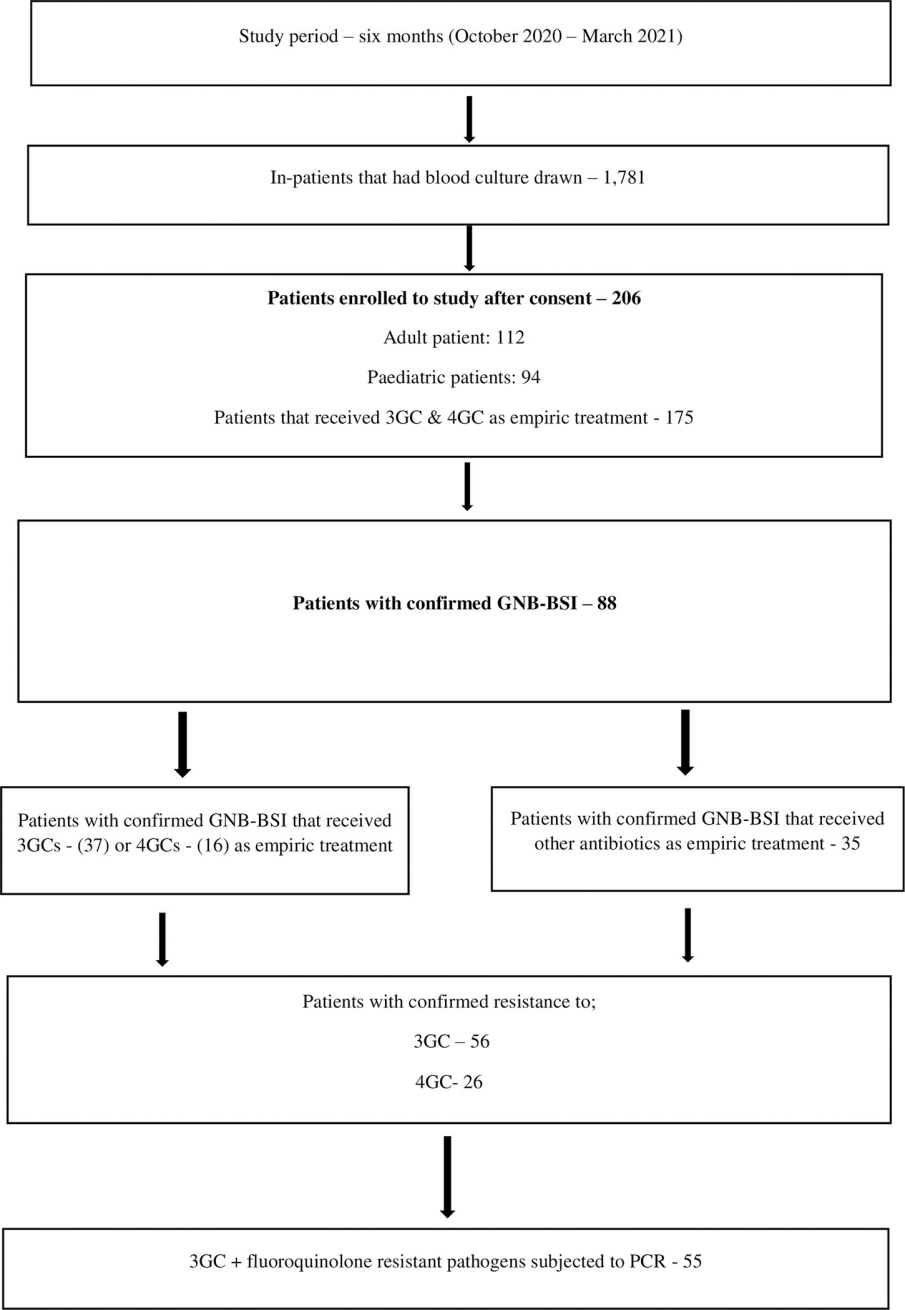
Flowchart of the enrolled patients and selection criteria.

Two hundred and six patients met the inclusion criteria and were enrolled to the study. Seventy-five percent had fever prior to admission, while 25% developed fever 48 hours post-admission. Only one patient among those that developed fever 48 hours post admission had a culture positive *Acinetobacter baumannii* BSI from a burns patient. The *Acinetobacter baumannii* isolated from this patient was MDR with resistance to both 3GCs and 4GCs respectively. Fifteen patients had a history of hospital admission in the last 30 days, and only one patient had received ceftriaxone during that admission. Of those that had history of admission in the last 30 days, only one patient had a culture positive *E*. *coli* BSI, an isolate that was susceptible to both 3GCs and 4GCs. Forty-three percent (88/206) of the enrolled patients had a confirmed GNB-BSI of which 42% and 18% received 3GCs and 4GCs as an empiric treatment, respectively. Notably, 68% of the patients that received 3GCs as empiric treatment had confirmed 3GC resistant pathogens (Fig 1).

Fourth generation cephalosporins (4GCs) were mainly prescribed in the NICU and other paediatric wards. Other antibiotics given as empiric treatment were ciprofloxacin and imipenem. Tuberculosis (TB) fixed-dose combination (FDC) with two or more anti-TB drugs, penicillin, metronidazole and co-trimoxazole were also commonly prescribed in combination with 3GCs. Co-morbidities in adult patients are listed in Table 1.

**Table 1:** The age and gender distribution and the co-morbidities of adult patients with confirmed GNB-BSI.

Characteristics	Female		Male		Total	
Age categories	Proportion (%)	95% CI	Proportion (%)	95% CI	Proportion (%)	95% CI
Neonates	53	30–75	47	27–69	**26**	**17–36**
Paediatrics	53	30–75	47	25–70	**20**	**13–31**
Adults	34	22–49	65	51–78	**54**	**43–64**
**Total**	**43**	**32–54**	**57**	**46–68**	**-**	**-**
**Co-morbidities in adult patients**	**Proportion**					
HIV	**55%**					
HIV co-infection with PTB	**7%**					
Diabetes	**6%**					
Hypertension (HTN)	**8%**					
Renal failure/chronic kidney disease (CKD)	**6%**					

Abbreviations: CI–confidence interval, HIV–Human immunodeficiency virus, PTB–Pulmonary tuberculosis

*HIV status was only collected in the adult population and only 29 patients disclosed their HIV status. The HIV proportion was calculated based on the total number of patients that disclosed their status.

Patients with confirmed GNB-BSI were categorized as neonates (≤ 28 days), paediatrics (≥ 29 days to < 16 years) and adults (≥16 years). There were relatively more adult cases than neonatal and paediatric cases, and the males were more represented than females (Table 1).

Out of the 88 GNB isolated, 77% were Enterobacterales (*E*. *coli*, *Klebsiella pneumoniae*, *Klebsiella aerogenes*, *Citrobacter freundii* and *Proteus mirabilis*), 15% *Acinetobacter baumannii* and 8% *Pseudomonas aeruginosa* respectively. *Escherichia coli* was the most prevalent cause of BSI, followed by *Klebsiella pneumoniae* (Table 2).

**Table 2:** Distribution of GNB isolated from blood cultures at UTH (2020 to 2021).

Pathogen	Proportion (%)	95% CI
*Escherichia coli*	42	32–52
*Klebsiella pneumoniae*	30	19–28
*Acinetobacter baumannii*	15	8–23
*Pseudomonas aeruginosa*	8	4–17
*Klebsiella aerogenes*	3	1–10
*Citrobacter freundii*	1	0–7
*Proteus mirabilis*	1	0–7

**Abbreviations:** CI–confidence interval

Table 3 highlights the GNB distribution per ward, with *E*. *coli* mainly being isolated in internal medicine (56%), *Klebsiella pneumoniae* in a neonatal intensive care unit (NICU) (42%), *Acinetobacter baumannii* in the surgical/burns unit (30%) and *Pseudomonas aeruginosa* in the renal unit (71%). Of note is the high prevalence of MDR GNB and the presence of possible XDR and PDR isolates in our study population. *E*. *coli* (42%) and *Klebsiella pneumoniae* (31%*)* had the most MDR isolates.

**Table 3:** Distribution of GNB per ward and MDR, XDR and PDR data.

	*E*. *coli*	*Klebsiella pneumoniae*	*Klebsiella aerogenes*	*Citrobacter freundii*	*Proteus mirabilis*	*Acinetobacter baumannii*	*Pseudomonas aeruginosa*	Total % (n)
Ward								
	**Frequency**							
I-MED	**21**	7	1	1	1	3	0	39% (34)
AICU	**4**	0	0	0	0	1	0	6% (5)
NICU	5	**12**	1	0	0	1	1	23% (20)
PICU	1	0	0	0	0	**2**	0	3% (3)
ADM	**4**	2	0	0	0	1	1	9% (8)
SURG	2	2	0	0	0	**4**	0	9% (8)
RENAL	0	3	1	0	0	1	**5**	11% (10)
**AMR Type**								
	**Frequency**							**Total % (n)**
MDR	31	23	2	1	1	12	3	83% (73)
Possible XDR	9	9	2	1	1	8	3	38% (33)
Possible PDR	0	0	1	0	0	2	1	5% (4)

**Wards: I-MED**–Internal Medicine, **AICU**–Adult Intensive Care Unit, **NICU**–Neonatal Intensive Care Unit, **PICU**–Paediatric Intensive Care Unit, **ADM**–Admission ward, **SURG**–Surgical

**Antibiotic resistance abbreviations: MDR**–Multidrug resistance: resistance to at least one agent in three or more antibiotic classes, **XDR**–Extensively drug resistance: resistance to at least one agent in all but two or fewer antimicrobial categories, **PDR–**Pan-drug resistance: resistance to all agents in all antimicrobial categories.

**Fig 2:**
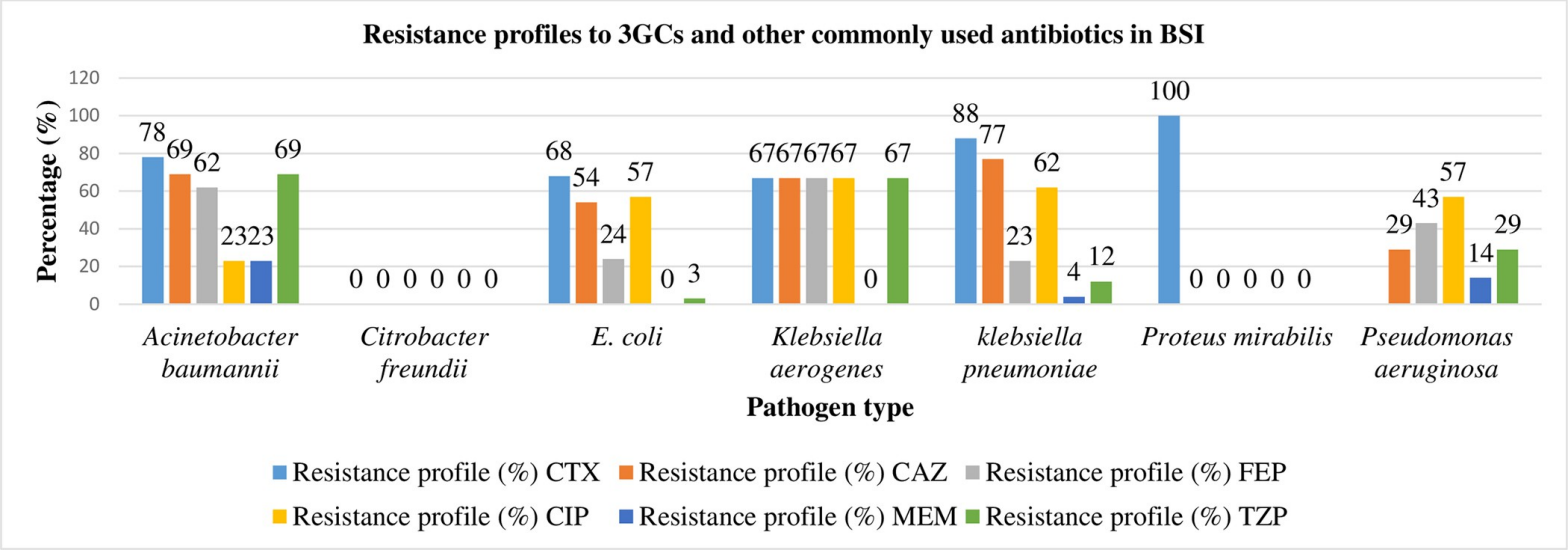
Resistance profiles of GNB to 3GCs and other commonly used antimicrobials in the treatment of BSI.

Antibiotic susceptibility patterns for all the GNB (n = 88) are shown in Table 4 and Fig 2. Resistance to cefotaxime was highest in *Klebsiella pneumoniae* (88%), *Acinetobacter baumannii* (78%), *E*. *coli* (68%) and *Klebsiella aerogenes* (67%). Resistance to ceftazidime in *Pseudomonas aeruginosa* isolates was 29%. Notable was high resistance to ciprofloxacin (64%), with *Klebsiella pneumoniae* and *Klebsiella aerogenes* having the highest ciprofloxacin resistance. *Acinetobacter baumannii* had the highest resistance to beta-lactam/beta-lactamase inhibitor (BL/BLI) (piperacillin-tazobactam). Carbapenem (meropenem) resistance was observed in three *Acinetobacter baumannii*, two *Pseudomonas aeruginosa* and one *Klebsiella pneumoniae* isolate, mostly from NICU 50% (3/6), renal unit 33% (2/6) and admission ward 17% (1/6).

**Table 4:** Antimicrobial susceptibility results (RIS %) to all the tested antimicrobials.

Antibiotics class			Susceptibility results		
	Number tested	Antibiotic	Resistance % (n)	Intermediate % (n)	Susceptible % (n)
**Beta-lactams**					
Penicillin	38	AMP	89% (34)	0	11% (4)
Beta-lactam combination agents	80	TZP	21% (17)	18% (14)	61% (49)
	67	AMC	37% (25)	29% (19)	34% (23)
	77	SAM	75% (58)	4% (3)	21% (16)
Cephamycins	67	FOX	8% (5)	1% (1)	91% (61)
2^nd^ generation cephalosporins	67	CXM	78% (52)	6% (4)	16% (11)
3^rd^ generation cephalosporins	81	CXT	75% (61)	3% (2)	22% (18)
	88	CAZ	60% (53)	2% (2)	38% (33)
4^th^ generation cephalosporins	88	FEB	32% (28)	18% (16)	50% (44)
Monobactams	74	ATM	46% (34)	1% (1)	53% (39)
**Aminoglycosides**					
	88	AMK	15% (13)	2% (2)	83% (73)
	88	GEN	55% (48)	1% (1)	44% (39)
**Quinolones**					
	88	CIP	64% (56)	2% (2)	34% (30)
**Folate pathway antagonist**					
	81	SXT	90% (73)	0	10% (8)
**Carbapenems**					
	88	MEM	7% (6)	1% (1)	92% (81)
**Nitrofurans**					
	67	NIT	33% (22)	15% (10)	52% (35)

**Abbreviations**: **RIS**–Resistant, Intermediate, Susceptible, **AMP**–Ampicillin, **TZP**–Piperacillin-Tazobactum, **AMC**–Amoxicillin-clavulanate, **SAM**–Ampicillin–Sulbactam, **FOX**–Cefoxitin, **C**MX–Cefuroxime, **CTX**–Cefotaxime, **CAZ–**Ceftazidime, **FEP**–Cefepime, **ATM**–Aztreonam, **AMK**–Amikacin, **GEN**–Gentamicin, **CIP**–Ciprofloxacin, **SXT**–Co-trimoxazole, **MEM**–Meropenem, **NIT**–Nitrofurantoin

In order to detect ESBLs, *amp*C and PMQR resistant gene determinants, a total of 55 GNB resistant to 3GCs and fluoroquinolones were subjected to PCR. Seventy-six per cent (42/55) were ESBL-producers, of which 50% were *Klebsiella pneumoniae*, 41%, *E*. *coli*, 5%, *Klebsiella aerogenes* and 2%, *Proteus mirabilis* and *Pseudomonas aeruginosa*, respectively. Those that harboured the *amp*C resistant gene were three *Klebsiella pneumoniae* and one *Pseudomonas aeruginosa*, all of which had ESBL resistance genes and were resistant to cefoxitin. PMQR determinants *qnr*A, *qnr*B and *qnrS* were confirmed in some GNB, with *qnr*A being the most prevalent. A few other multiple gene combinations were also confirmed in some isolates (Table 5).

**Table 5:** ESBL, *amp*C and PMQR resistant genes determinants.

ESBL, *amp*C and PMQR resistant genes determinants		
Isolates tested– 55		
**ESBL Type**	**Proportion% (n)**	**95% CI**
*bla*_SHV_	51% (28)	38–64
*bla*_CTX-M_	47% (26)	34–61
*bla*_TEM_	45% (25)	33–59
*bla*_CTX-M_ & *bla*_TEM_	31% (17)	20–45
*bla*_SHV_ & *bla*_TEM_	31% (17)	20–45
*bla*_CTX-M_ & *bla*_SHV_	27% (15)	16–40
*bla*SHV & *bla*CTX-M & *bla*TEM	20% (11)	11–32
*bla*SHV & *bla*CTX-M & *bla*TEM & *amp*C	2% (1)	0.2–12
***amp*C β-lactamase**		
*Amp*C	7% (4)	2–18
**PMQR determinants**		
*qnrA*	25% (14)	15–38
*qnrB*	20% (11)	11–32
*qnrS*	12% (7)	6–24
*qnrA* & *qnrB*	5% (3)	1–15
*qnrB & qnrS*	4% (2)	0.8–13
*qnrA* & *qnrS*	2% (1)	0.2–12

**PMQR**–Plasmid Mediated Quinolone Resistance, CI- Confidence interval

In order to determine potential risk factors associated with treatment outcome, the association between the dichotomous outcome variable (death/recovery) and explanatory variable were analysed using the Fisher’s exact test. All variables with a p-value <0.20 (Table 6) were selected in the univariable analysis to build the multivariable logistic regression. Collinearity was observed between the "age category” and “length of hospital stay” (p-value = < 0.001) and between the "age category” and “empirical treatment” (p-value = < .001).

**Table 6:** Results of the univariate analysis in the Fisher’s exact test, with treatment outcome (death or recovery) as an outcome variable for patients at UTH.

Variables	Level	The proportion of Death %	95% CI	P-value
Age Category	Neonates	9.5	2.3–32.4	0.001
	Paediatrics	41.2	20.3–65.8	
	Adults	54.7	39.3–69.3	
Duration of stay	Short (≤25 days)	47.6	35.4–60.1	0.006
	Long (>25days)	11.7	2.7–38.4	
Empirical Treatment	3GC	56.7	40.2–71.9	0.007
	4GC	12.5	2.9–40.3	
	Other	33.3	17.9–53.4	
Sex	Male	33.3	20.9–48.8	0.178
	Female	48.6	32.3–65.1	
Ward-Type (Department)	Medical	50.0	33.3–66.6	0.002
	AICU	0	-	
	NICU	10.0	2.4–33.7	
	PICU	100	-	
	Admissions	42.8	12.7–79.5	
	Surgery	100	-	
	Renal	50.0	18.1–81.8	
Bacteria species	*E*. *coli*	41.1	26.5–58.6	0.144
	*Acinetobacter baumannii*	70.0	35.4–90.8	
	*Klebsiella Pneumoniae*	27.3	12.4–49.9	
	Others*	33.3	12.3–64.1	

*Pseudomonas aeruginosa, *Proteus mirabilis*, and *Klebsiella aerogenes* were classified as "others" because there were few observations.

Results from the multivariable logistic regression analysis are shown in Table 7. As shown, "Type of Bacterial species", "Empirical Treatment", and "Sex of Participants” were explanatory variables retained in the final model. The logistic regression model adequately fitted the data (Hosmer and Lemeshow test: Pearson chi2 (14) = 17.38; Prob > chi2 = 0.24), with reasonable explanatory power, with ROC areas around 0.76 (a ROC area of 0.50 indicates no explanatory power).

**Table 7:** Multivariable logistic regression model for risk factors associated with treatment outcome (death/recovery) in patients at UTH.

Variables	Level	Odds Ratio	P-values	95%CI
Bacteria species	*E*. *coli*	Baseline		
	*Acinetobacter baumannii*	3.8	0.120	0.7–20.8
	*Klebsiella Pneumoniae*	0.9	0.950	0.3–3.5
	Others	1.1	0.933	2.3–5.0
Empirical Treatment	4GC	Baseline		
	Other	3.1	0.227	0.5–20.1
	3GC	13.4	0.006	2.1–84.8
Sex	Male	Baseline		
	Female	3.3	0.040	1.0–10.1
Constant		0.02	0.005	0.001–0.29

In comparison to *E*. *coli* as a causative agent, the odds of death were significantly higher among patients infected with *Acinetobacter baumannii* (OR = 3.8) than *Klebsiella pneumoniae* (OR = 0.9) or other causative agents (OR = 1.1). The odds of death were significantly higher among patients receiving 3GCs (OR = 13.4) or other types of antibiotics (OR = 3.1) than those receiving 4GCs as empiric treatment. The odds of death in female patients compared to male patients was 3.3.

## 4. Discussion

Our findings suggest that the prevalence of BSI caused by GNB resistance to 3GCs was high, and the use of 3GCs as empiric treatment negatively affected the outcome of admitted patients, as is seen by the high mortality in the patients that received 3GCs as empiric treatment.

The number of patients that received 3GCs as empiric treatment and were later confirmed to have BSI caused by 3GC resistant GNB was equally high at 68%. This finding indicates a high prevalence of inappropriate empirical treatment at our referral hospital, similar to a previous study done three years ago at the same hospital, that found 67% of antimicrobial orders to be inappropriately prescribed, with 3GCs being the most prescribed (83%) [18]. Inappropriate prescribing in this study, could be attributed to the lack of an updated antibiogram and the microbiology diagnostic challenges as result of reagent stock outs [18]. In 2022, the hospital released and launched an updated antibiogram coupled with a mobile application which is currently in use [21]. Contrary to our findings, a study done in Cape Town, South Africa, recorded a lower prevalence of inappropriate empiric treatment (30.6%) [32].

Inappropriate empirical antimicrobial therapy has been shown to be associated with increased mortality in young children and neonates, thereby highlighting the importance of appropriate empirical antibiotic recommendations [33]. However, the lack of AMR structured surveillance and reporting that is required to support the most appropriate local treatment guidelines and the lack of rapid diagnostic tests in our setting delays the de-escalation or commencement of target-specific antibiotics. This has led to the prolonged use of broader spectrum antibiotics and inappropriate regimens, a practice known to create selective antibiotic pressure, thereby increasing resistance among pathogens [16, 33, 34].

Given the extensive dependence on beta-lactams, especially ceftriaxone, for management of BSI in our setting, 3GC resistant Enterobacterales are of particular concern as the knowledge of AMR patterns, and effective surveillance has significant implications on patient management and outcome. Notable is that despite the high prevalence of 3GC resistance in our setting, as was also noted in previous studies [17, 18, 21], there has limited guidance based on institutional surveillance and formulation of institution/unit-specific antibiogram guidelines until recently in 2022 [21]. A recent multi-facility cross-sectional study that reviewed and analysed the antibiotic prescribing patterns in adult patients at primary healthcare hospitals in Zambia found ceftriaxone (20.3%) to be the most prescribed antibiotic [35]. Although lower than what was previously recorded at the UTH, most primary healthcare hospitals in Zambia lack fully functional diagnostic laboratories that perform culture and antimicrobial susceptibility testing, hence rely on empiric treatment with no further guidance on target-specific antimicrobial treatment or de-escalation. This use of 3GCs in primary care facilities promotes the emergence of 3GC-resistant pathogens thereby further limiting treatment options for patients being referred to tertiary hospital where 3GCs are widely used as empiric treatment [36].

Similar to the findings at the UTH and the primary care facilities in Lusaka [35, 36], another study in Zambia that reviewed antibiotic use and stewardship indicators in the first- and second-level hospitals in ten provinces at ten different hospitals found the prevalence of antibiotic use among the in-patients to be at 59% with a high rate of empiric prescribing (97%), of which ceftriaxone was 36% of all antibiotics prescribed [37]. Low compliance to the national standard treatment guidelines (STGs), low justified antibiotic use at 16% and only 3% of the treatment having been guided by microscopy, culture and sensitivity (MCS). Practices that drive the emergence of AMR [37].

The paediatric age group with the most infections was ≤12 months; this was comparable to studies in South Africa [32] but contrary to a study in Tanzania which found children above one year of age to be the most admitted with BSI [38]. Among other factors, an immature immune system in the age group ≤12 months is likely to contribute to the high prevalence of BSI seen in this study [39]. Prematurity and malnutrition are other factors associated with high BSI prevalence in this age group and are common in low-resource countries [17, 38]. The high prevalence of *E*. *coli* and *Klebsiella pneumoniae* observed in this study is comparable to findings in a previous Zambian study at the same hospital [40] and other studies done in, Tanzania, Ethiopia, South Africa and Botswana [41–43]. Only one MDR *Acinetobacter baumannii* was associated with HAI in a burns patient. Notably is the high prevalence of *Klebsiella pneumoniae-*BSI in NICU, similar to the findings in a previous study in Zambia [17], South Africa [44, 45] and Malawi [46]. This finding can result from extensive physical contact, vertical transmission and poor infection control measures, posing treatment challenges due to AMR and limited permitted antibiotics in this age group [47].

A study that reviewed the prevalence and outcomes of MDR-BSIs found that patients admitted with MDR-BSIs were more likely to receive inappropriate empiric treatment, leading to longer ICU LOS, higher treatment costs and mortality [48]. Resistance to 3GCs was also seen in the community on-set infections, replicating the findings of a Malawian study that although the prevalence of community-acquired (CA) ESBL-E was low (16.67%), they confirmed the existence of ESBL-E in patients that had no history of hospital admission in the last three months [49]. This finding has been observed over the years in high-, middle- and low-income countries [50–55]. Comparable to our findings, a seven-year Korean study and another study in Taiwan observed increased community on-set ESBL-producing *E*. *coli* infections [56, 57]. This was attributed to the spread of CTX-M type ESBLs in the community worldwide, especially in *Escherichia coli* [56]. In our setting, the high prevalence of CA AMR infections could result from the over-the-counter non-prescribed purchase of oral antibiotics, non-compliance to treatment duration, and other environmental and food animal sources [58, 59].

The high resistance to 3GCs (75%) observed in this study confirmed an increase from what was previously observed at the same hospital in 2015 to 207 [40]. This finding confirms the growing problem of 3GC resistance in our hospital, thereby emphasizing the need for improved rapid laboratory diagnostic capacity and AMR screening with decent turn-around time, continuous hospital-based surveillance and yearly antibiogram revision. Our findings replicated a two-decade study in Malawi that found a marked increase in resistance to first-line beta-lactam antibiotics and a systematic review in sub-Saharan countries that found the prevalence of 3GC resistance in *E*. *coli*-BSI greater than estimates from high-income countries, with *E*. *coli* being the leading cause of death in 2019 [3, 47, 60, 61]. The growing trend of intermediate resistance and full resistance in other antibiotic classes such as BL/BLI, fluoroquinolones and carbapenems further complicate the treatment options for patients presenting with BSI. This is more so for carbapenems, the treatment of choice for MDR and ESBL-producing-Enterobacterales BSI [14, 15].

ESBL detection in GNB is considered an essential marker for treatment outcomes. The importance of studying the ESBL-encoding genes (*bla* genes) and characteristics of their location on mobile genetic elements such as plasmids and integrons responsible for horizontal transfer between species, other Enterobacterales and GNB cannot be overemphasized [62]. Most studies in Zambia on the subject of 3GC resistance and ESBLs were based on characterisation of phenotypic resistance patterns rather than the genes responsible [17, 40]. This undermines the understanding of ESBL gene diversity implicated in HCAI and CAI caused by ESBL-producing strains [62]. The *bla*_SHV_ gene was the most prevalent among the *bla*genes detected in this study, compared to studies that found *bla*_CTX-M_ to be more prevalent in the developed world [63], Poland [64], and Eastern, Southern, Northern, Western, and Central African countries [65, 66]. An earlier study at UTH similarly found *bla*_*SHV*_ to be the predominant ESBL-encoding gene [67]. However, this previous study did not find *bla*_CTX-M_, the second-highest resistance gene observed in this study. This finding confirms the evolution of ESBL-encoding genes in our setting.

Bacteria that carry ESBL and *Amp*C genes often carry additional genes or mutations in genes that mediate resistance to a broad range of antibiotics [68]. This was observed in our findings of ESBLs and PMQR determinants *qnr*A, *qnr*B and *qnrS* co-existence, further limiting the treatment options for BSI. Of serious concern was the emergence of carbapenem resistance (7%). Currently, antibiotic options for carbapenem-resistant Enterobacterales (CRE) treatment are minimal and complex, with polymyxins, high-dose tigecycline, fosfomycin, next-generation aminoglycosides, and new BL/BLI as the mainstays of therapy [69]. These antibiotics are effective, but the need for new and effective anti-CRE therapies cannot be over-emphasized [70].

The LOS and outcome of in-patients depend on the hospital environment, the severity of the disease, and treatment efficiency and effectiveness [71]. In neonatal patients, the prolonged LOS could also be attributed to other factors such as prematurity and low birth weight while, co-morbidities such as HIV, HTN, diabetes, and kidney failure contribute to prolonged LOS and poor treatment outcomes in adult patients [72, 73]. The significantly high odds of death in patients that received 3GCs as empiric treatment compared to those that received 4GCs confirms the negative effect of delayed appropriate antimicrobial therapy in patients infected with MDR GNB [74]. Noteworthy prolonged LOS, high mortality and in-hospital costs have also been attributed to carbapenem resistant Enterobacterales CRE [75]. CRE was relatively low (7%) in this study.

This study provided information on epidemiological, clinical and microbiological features and the effects of antimicrobial resistance on the outcomes of BSIs at a referral hospital in Lusaka, Zambia. Information that can form a basis for surveillance and antimicrobial stewardship programs.

### 4.1 Study limitation

The findings in this study should be taken with caution because the study was limited to only one health facility, which was purposely selected. Further, the study was limited to only those patients whose blood samples were submitted to the Microbiological Laboratory for analysis which meant a possible exclusion of patients with blood infections but had no opportunity to have their blood tested. This could bias the study population and therefore reduces the external validity of the findings. Despite these limitations, the study has provided valuable insights into understanding the resistance pattern of GNB-BSI and the patient treatment outcomes.

## 5. Conclusions

The prevalence of BSI caused by GNB resistant to 3GCs was high, and the use of 3GCs as empiric treatment negatively affected the outcome of patients, as was seen by the high mortality in the patients that received 3GCs as empiric treatment. Notably was the high number of cases of inappropriate empirical treatment. Yearly antibiogram formulation and updates using locally generated antimicrobial susceptibility data, coupled with a well functional Antimicrobial stewardship (AMS) program will not only reduce the emergence of AMR and healthcare costs associated with inappropriate antimicrobial use and prolonged LOS, but will also improve patient clinical outcomes.

## Supporting information

S1 Table Primers and PCR condition used in this study. (TIF)

S1 Data Gram negative antimicrobial susceptibility inputs. (XLSX)

S2 Data Resistance genes analysis. (XLSX)

S3 Data Patient outcomes analysis. (XLSX)

S4 Data Statistical analysis for proportion of resistance genes. (DOCX)

S5 Data Statistical analysis for relationship between treatment and outcomes. (DOCX)
