# Supplementary figures and images for: Phenotypic and genotypic antibiotic susceptibility profiles of Gram-negative bacteria isolated from bloodstream infections at a referral hospital, Lusaka, Zambia
Source: PLOS Glob Public Health. 2023 Jan 31;3(1):e0001414. doi: 10.1371/journal.pgph.0001414 (PMC10021926; doi:10.1371/journal.pgph.0001414)

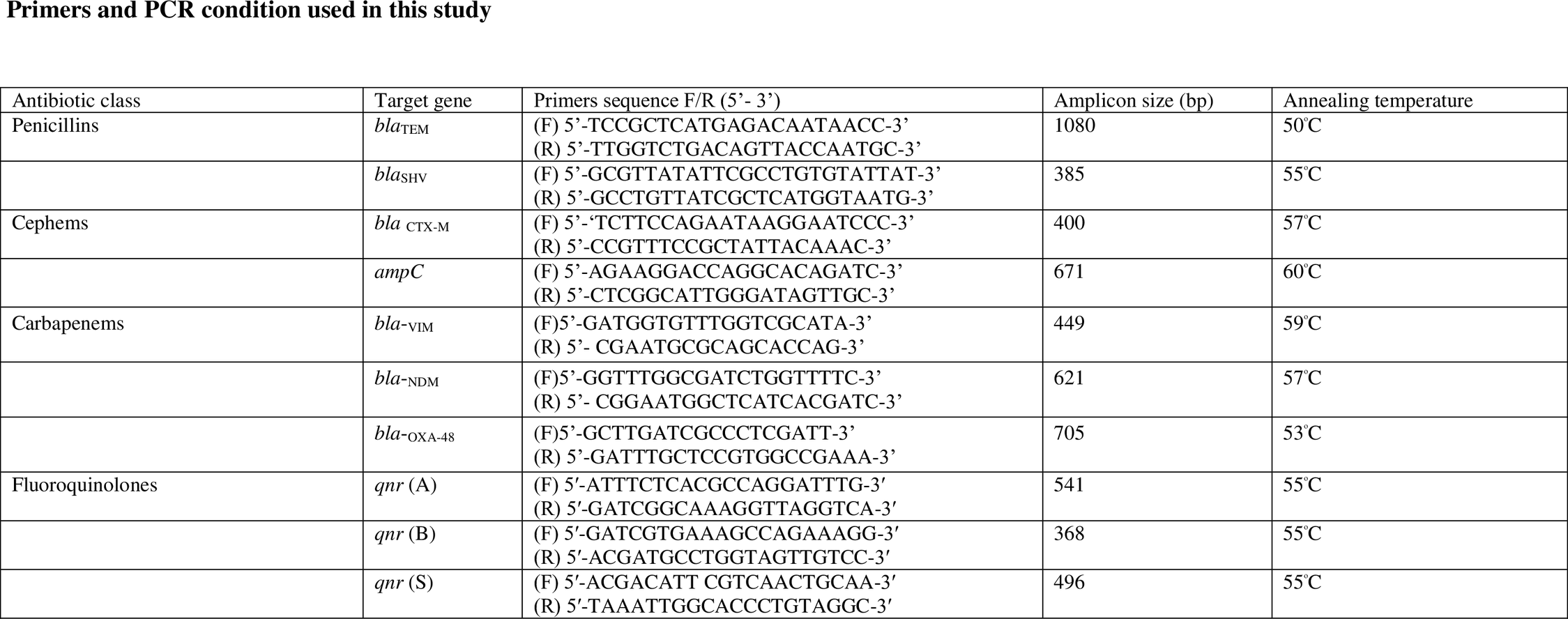

Supplement: S1 Table — (TIF) [file pgph.0001414.s001.tif]
