# Supplementary material for: Phenotypic and genotypic antibiotic susceptibility profiles of Gram-negative bacteria isolated from bloodstream infections at a referral hospital, Lusaka, Zambia
Source: PLOS Glob Public Health. 2023 Jan 31;3(1):e0001414. doi: 10.1371/journal.pgph.0001414 (PMC10021926; doi:10.1371/journal.pgph.0001414)
Supplement: S4 Data — (DOCX) [file pgph.0001414.s005.docx]

proportion ctx-m

Proportion estimation Number of obs = 55

--------------------------------------------------------------

| Logit

| Proportion Std. Err. [95% Conf. Interval]

-------------+------------------------------------------------

Ctx-m |

0 | .5272727 .0673196 .3935814 .6571639

1 | .4727273 .0673196 .3428361 .6064186

Tem

. proportion tem

Proportion estimation Number of obs = 55

--------------------------------------------------------------

| Logit

| Proportion Std. Err. [95% Conf. Interval]

-------------+------------------------------------------------

esbl |

0 | .5454545 .0671408 .4108138 .6737611

1 | .4545455 .0671408 .3262389 .5891862

--------------------------------------------------------------

. proportion Shv

Proportion estimation Number of obs = 55

--------------------------------------------------------------

| Logit

| Proportion Std. Err. [95% Conf. Interval]

-------------+------------------------------------------------

esbl |

0 | .4909091 .0674088 .3595926 .6234922

1 | .5090909 .0674088 .3765078 .6404074

proportion qnrA

Proportion estimation Number of obs = 55

--------------------------------------------------------------

| Logit

| Proportion Std. Err. [95% Conf. Interval]

-------------+------------------------------------------------

qnrA |

0 | .7454545 .058737 .611569 .844896

1 | .2545455 .058737 .155104 .388431

. proportion qnrB

Proportion estimation Number of obs = 55

Logit

Proportion Std. Err. [95% Conf. Interval]

qnrB

0 .8 .053936 .6705005 .8871685

1 .2 .053936 .1128315 .3294995

**proportion qnrS**

Proportion estimation Number of obs = 55

--------------------------------------------------------------

| Logit

| Proportion Std. Err. [95% Conf. Interval]

-------------+------------------------------------------------

qnrs |

0 | .8727273 .0449392 .7529007 .9391429

1 | .1272727 .0449392 .0608571 .2470993

-------------------------------------------------------------

. proportion ampc

Proportion estimation Number of obs = 55

--------------------------------------------------------------

| Logit

| Proportion Std. Err. [95% Conf. Interval]

-------------+------------------------------------------------

ampc |

0 | .9272727 .0350164 .818248 .9730523

1 | .0727273 .0350164 .0269477 .181752

proportion qnra_code qnrb_code qnrs_code ctxm_code tem_code shv_code ampc_code ctxm_tem_shv_amc c

> txm_tem_shv ctxm_tem_amc ctxm_tem_amc ctxm_shv_amc tem_shv_amc ctxm_tem ctxm_shv ctxm_ampc shv_te

> m shv_ampc tem_ampc

Proportion estimation Number of obs = 55

Logit

Proportion Std. Err. [95% Conf. Interval]

qnra_code

0 .7454545 .058737 .611569 .844896

1 .2545455 .058737 .155104 .388431

qnrb_code

0 .8 .053936 .6705005 .8871685

1 .2 .053936 .1128315 .3294995

qnrs_code

0 .8727273 .0449392 .7529007 .9391429

1 .1272727 .0449392 .0608571 .2470993

ctxm_code

0 .5272727 .0673196 .3935814 .6571639

1 .4727273 .0673196 .3428361 .6064186

tem_code

0 .5454545 .0671408 .4108138 .6737611

1 .4545455 .0671408 .3262389 .5891862

shv_code

0 .4909091 .0674088 .3595926 .6234922

1 .5090909 .0674088 .3765078 .6404074

ampc_code

0 .9272727 .0350164 .818248 .9730523

1 .0727273 .0350164 .0269477 .181752

ctxm_tem_shv_amc

0 .9818182 .0180158 .8771405 .9975576

1 .0181818 .0180158 .0024424 .1228595

ctxm_tem_shv

0 .8 .053936 .6705005 .8871685

1 .2 .053936 .1128315 .3294995

ctxm_tem_amc

0 .9636364 .0252411 .8621164 .9911751

1 .0363636 .0252411 .0088249 .1378836

ctxm_shv_amc

0 .9818182 .0180158 .8771405 .9975576

1 .0181818 .0180158 .0024424 .1228595

tem_shv_amc

0 .9636364 .0252411 .8621164 .9911751

1 .0363636 .0252411 .0088249 .1378836

ctxm_tem

0 .6909091 .0623121 .5546252 .8004915

1 .3090909 .0623121 .1995085 .4453748

ctxm_shv

0 .7272727 .0600526 .5923822 .8303115

1 .2727273 .0600526 .1696885 .4076178

ctxm_ampc

0 .9636364 .0252411 .8621164 .9911751

1 .0363636 .0252411 .0088249 .1378836

shv_tem

0 .6909091 .0623121 .5546252 .8004915

1 .3090909 .0623121 .1995085 .4453748

shv_ampc

0 .9636364 .0252411 .8621164 .9911751

1 .0363636 .0252411 .0088249 .1378836

tem_ampc

0 .9454545 .0306209 .840532 .982759

1 .0545455 .0306209 .017241 .159468
