# Supplementary material for: Phenotypic and genotypic antibiotic susceptibility profiles of Gram-negative bacteria isolated from bloodstream infections at a referral hospital, Lusaka, Zambia
Source: PLOS Glob Public Health. 2023 Jan 31;3(1):e0001414. doi: 10.1371/journal.pgph.0001414 (PMC10021926; doi:10.1371/journal.pgph.0001414)
Supplement: S5 Data — (DOCX) [file pgph.0001414.s006.docx]

**Relationship Between Treatment and outcome (All treatment type)**

--------------------------------------------------------------

Over | Proportion Std. Err. [95% Conf. Interval]

-------------+------------------------------------------------

_3GC 1 |

0 | .3333333 .0687614 .2126467 .4806973 (Alive)

1 | .65625 .0853051 .4735098 .802075 (Dead)

-------------+------------------------------------------------

_4GC_2 |

0 | .2916667 .0663 .1785699 .438183 (Alive)

1 | .0625 .0434755 .014995 .2259765 (Dead)

-------------+------------------------------------------------

_Others_3 |

0 | .375 .0706166 .2477696 .5222084 (Alive)

1 | .28125 .0807522 .1501539 .4642748 (Dead)

Proportion estimation Number of obs = 37

_prop_1: ctx = 0

_prop_2: ctx = 2

_prop_3: ctx = 3

0: patient_outcome = 0

1: patient_outcome = 1

**Relationship Between ctx and outcome**

--------------------------------------------------------------

Over | Proportion Std. Err. [95% Conf. Interval]

-------------+------------------------------------------------

Resist_1 |

0 | .6875 .1196784 .4154963 .871938 (Alive)

1 | .6666667 .1054093 .4331864 .8395868 (Dead)

-------------+------------------------------------------------

Inter2 |

0 | .1875 .1007782 .0568956 .4688616 (Alive)

1 | .2380952 .0952381 .0972729 .4754195 (Dead)

-------------+------------------------------------------------

_Suscep_3 |

0 | .125 .0853913 .0284904 .4103457 (Alive)

1 | .0952381 .0656383 .0219629 .3303968 (Dead)

--------------------------------------------------------------

\

proportion ctx2 if type_empirical==0, over(patient_outcome)

Proportion estimation Number of obs = 37

_prop_1: ctx2 = 0

_prop_2: ctx2 = 3

0: patient_outcome = 0

1: patient_outcome = 1

--------------------------------------------------------------

Over | Proportion Std. Err. [95% Conf. Interval]

-------------+------------------------------------------------

**Resistant (n=73)**  |

Alive | **.875** .0853913 .5896543 .9715096

**Dead** | .**90**47619 .0656383 .6696032 .9780371

-------------+------------------------------------------------

**Susceptible (n=7)**  |

**Alive** | .125 .0853913 .0284904 .4103457

Dead | .0952381 .0656383 .0219629 .3303968

**Relationship between Resistance to Cxt and patient outcome**

. tab ctx2 patient_outcome, exact

| Patient_outcome

ctx2 | 0 1 | Total

-----------+----------------------+----------

0 | 44 29 | 73

3 | 4 3 | 7

-----------+----------------------+----------

Total | 48 32 | 80

Fisher's exact **= 1.000**

1-sided Fisher's exact = 0.586

**Duration**

mean durationo0admission, over(ctx2)

Mean estimation Number of obs = 80

0: ctx2 = 0

3: ctx2 = 3

---------------------------------------------------------------------

Over | Mean Std. Err. [95% Conf. Interval]

**--------------------+------------------------------------------------**

**Durationo0admission |**

0 **| 16.60274** 1.108247 14.39683 18.80865

3 **| 11.71429** 2.714286 6.311635 17.11694

---------------------------------------------------------------------

**Tuesday, August 09, 2022**

mean durationo0admission, over(type_empirical)

Mean estimation Number of obs = 80

0: type_empirical = 0

1: type_empirical = 1

2: type_empirical = 2

Over Mean Std. Err. [95% Conf. Interval]

durationo0admission

0 11.86486 .7741722 10.32391 13.40582

1 27.125 2.128918 22.88749 31.36251

2 15.59259 1.855353 11.89961 19.28558
